# Hyaluronic Acid-Modified Cisplatin-Encapsulated Poly(Lactic-co-Glycolic Acid) Magnetic Nanoparticles for Dual-Targeted NIR-Responsive Chemo-Photothermal Combination Cancer Therapy

**DOI:** 10.3390/pharmaceutics15010290

**Published:** 2023-01-14

**Authors:** Huai-An Chen, Yu-Jen Lu, Banendu Sunder Dash, Yin-Kai Chao, Jyh-Ping Chen

**Affiliations:** 1 Department of Chemical and Materials and Materials Engineering, Chang Gung University, Kwei-San, Taoyuan 33302, Taiwan; 2 Department of Neurosurgery, Chang Gung Memorial Hospital at Linkou, Chang Gung University School of Medicine, Kwei-San, Taoyuan 33305, Taiwan; 3 Division of Thoracic Surgery, Chang Gung Memorial Hospital at Linko, Chang Gung University School of Medicine, Taoyuan 33305, Taiwan; 4 Craniofacial Research Center, Chang Gung Memorial Hospital at Linkou, Kwei-San, Taoyuan 33305, Taiwan; 5 Research Center for Food and Cosmetic Safety, College of Human Ecology, Chang Gung University of Science and Technology, Taoyuan 33302, Taiwan; 6 Department of Materials Engineering, Ming Chi University of Technology, Tai-Shan, New Taipei City 24301, Taiwan

**Keywords:** nanomedicine, chemotherapy, photothermal therapy, magnetic nanoparticles, cancer therapy

## Abstract

Combination chemo-photothermal therapy with nanomaterials can reduce the dose of chemotherapeutic drugs required for effective cancer treatment by minimizing toxic side effects while improving survival times. Toward this end, we prepare hyaluronic acid (HA)-modified poly(lactic-co-glycolic acid) (PLGA) magnetic nanoparticles (MNP) for the CD44 receptor-mediated and magnetic field-guided dual-targeted delivery of cisplatin (CDDP). By co-encapsulating the CDDP and oleic acid-coated iron oxide MNP (IOMNP) in PLGA, the PMNPc was first prepared in a single emulsification/solvent evaporation step and successively surface modified with chitosan and HA to prepare the HA/PMNPc. Spherical HA/PMNPc nanoparticles of ~300 nm diameter can be prepared with 18 and 10% (*w*/*w*) loading content of CDDP and IOMNP and a pH-sensitive drug release to facilitate the endosomal release of the CDDP after intracellular uptake. This leads to the higher cytotoxicity of the HA/PMNPc toward the U87 glioblastoma cells than free CDDP with reduced IC50, a higher cell apoptosis rate, and the enhanced expression of cell apoptosis marker proteins. Furthermore, the nanoparticles show the hyperthermia effect toward U87 after short-term near-infrared (NIR) light exposure, which can further elevate the cell apoptosis/necrosis rate and upregulate the HSP70 protein expression due to the photothermal effects. The combined cancer therapeutic efficacy was studied in vivo using subcutaneously implanted U87 cells in nude mice. By using dual-targeted chemo-photothermal combination cancer therapy, the intravenously injected HA/PMNPc under magnetic field guidance and followed by NIR laser irradiation was demonstrated to be the most effective treatment modality by inhibiting the tumor growth and prolonging the survival time of the tumor-bearing nude mice.

## 1. Introduction

Cisplatin (cis-diamminedichloro-platinum II, CDDP) was initially discovered from Rosenberg’s electric field experiment in 1965, where its ability to prevent the growth of *Escherichia coli* was found [1]. Later, the drug was further recognized for its anti-neoplastic and cytotoxic effects on cancer cells, and it was approved by the U.S. Food and Drug Administration (FDA) for clinical use in 1978 [2]. Nowadays, CDDP is widely used in the treatment of a variety of cancers, including testicular, ovarian, head and neck, and lung cancers [3]. The cytotoxic mechanism of CDDP toward cancer cells was linked to its ability to interact with the purine bases in DNA, which leads to adduct formation and interference with DNA repair during DNA damage and eventually leads to cell apoptosis or programmed cell death [4]. For brain cancer treatment, cisplatin entered clinical trials for glioblastoma and high-grade gliomas brain tumor patients and was found to be effective for suppressing tumor growth and prolonging the patient median survival time [5]. However, the clinical use of CDDP is restricted by the severity of the side effects from drug toxicity, such as gastrointestinal toxicity, nephrotoxicity (kidney damage), and hepatotoxicity (liver damage) [6,7]. Furthermore, drug resistance remains a factor that hinders the clinical usefulness of CDDP, where many patients may initially show a good reaction but eventually may relapse and develop resistance [8]. Consequently, several strategies have emerged to reduce the side effects while retaining the potency of CDDP, such as the addition of antioxidants and anti-inflammatory agents [9,10], nanomedicine [11,12], or combination cancer therapy [13,14].

Nanomedicine using nanoparticle-based drug delivery systems has great potential in cancer therapy, including the intracellular delivery of CDDP [15]. By encapsulating CDDP in biodegradable polymeric nanoparticles, a desired drug release profile that is adaptable for the treatment could be achieved, which can also avoid the side effects without reducing the efficiency for cancer treatments [16,17]. Recently, magnetic nanoparticles (MNP), especially iron oxide MNP (IOMNP), were used extensively in various fields, such as drug delivery [18], biosensing [19], energy storage [20], environmental modification [21], and magnetic fluids [22]. For biomedical applications, the MNP can be facilely used in magnetic resonance imaging (MRI) [23], for magnetic targeting by magnetic field guidance to a localized site [24], and as a photothermal agent in photothermal cancer therapy after exposure to near-infrared (NIR) light [25]. In addition to magnetic targeting in cancer therapy, the functionality of drug-loaded MNP could be further enhanced through surface modification with ligand molecules to target cancer cells [26]. Undoubtedly, this ligand-mediated active targeting strategy, offered by the highly specific binding between the ligand-conjugated nanoparticles and cancer cells overexpressing receptor toward the ligand, can increase the intracellular uptake efficiency of the drug-loaded nanocarrier and raise the therapeutic efficacy [27].

The nanoparticles based on poly(lactic-co-glycolic acid) (PLGA) can be used as a tumor-targeting nano-platform for the delivery of different therapeutic agents in cancer therapy [28]. Considering cancer photothermal therapy, an NIR-responsive photothermal agent (e.g., gold nanoparticle (Au NP), poly(dopamine) (PDA), polypyrrole (PPy), IR780, and IR820) can absorb the photon energy and transform it to heat from the photothermal effect, which will raise the temperature of the tumor to cause tumor ablation [29]. Therefore, PLGA nanoparticles can entrap or be coated with these agents and modified with targeting ligands for targeted cancer therapy. An IR820-loaded PLGA nanoparticle system was evaluated for the photothermal therapy of triple-negative breast cancer in vitro and in vivo [30]. In a different study, magnetic PLGA nanocomposites were prepared by co-entrapping hydrophobic IOMNP and chemotherapeutic drugs, followed by modification with the transferrin receptor-binding peptide T7a for targeting cancer cells [31]. By combining magnetic guidance and active targeting, a dual-targeting strategy was used for the co-delivery of paclitaxel and curcumin in brain tumor therapy. Undoubtedly, the advanced design of PLGA-based nanomaterials could be facially achieved by co-loading chemotherapeutic agents and photothermal agents for combination chemo-photothermal cancer therapy [32]. Along this line, PLGA was used for co-loading docetaxel (DTX) [33]. The Au NP was deposited on the surface of a DTX-loaded PLGA nanoparticle, followed by decorating with a tumor-targeting peptide angiopep-2, to achieve active targeted drug delivery in combination chemo-photothermal therapy. Similarly, DTX-loaded PLGA nanoparticles were coated with PDA and modified with D-α-tocopherol polyethylene glycol 1000 succinate to inhibit the P-glycoprotein-mediated multidrug resistance. Both in vitro and in vivo studies indicated this nanoparticle can overcome multidrug resistance for chemo-photothermal synergistic antitumor therapy of breast cancer [34]. Another study also used PDA-modified doxorubicin-loaded PLGA nanoparticles for synergistic chemo-photothermal therapy [7]. Using PPy as a photothermal agent, a PLGA nanocomposite was synthesized by co-entrapping DTX and PPy, followed by surface modification with 2-deoxy-glucose-terminated PEG [35]. Endowed with the enhanced intracellular uptake efficiency by cancer cells, the nanocomposite showed similar photothermal properties of PPy and was used successfully for chemo-photothermal therapy against breast cancer cells. Finally, by entrapping IOMNP, IR780, doxorubicin, and perfluoropentane in PLGA nanoparticles, magnetically targeted nanocarriers for nanotheranostics could be designed with a photothermal-triggered drug release ability for imaging-guided chemo-photothermal therapy of breast cancer [36].

Hyaluronic acid (HA) is a natural polysaccharide found in the extracellular matrix. It is composed of repeating disaccharides of D-glucuronic acid and N-acetyl-D-glucosamine linked by alternating (1→3) and (1→4) linkages to form a polymer with up to 25,000 monomers. The HA is biodegradable and cytocompatible and will not elicit an inflammatory as well as immune response in the body [37]. Many nanoparticle-based drug delivery systems for cancer therapy use surface-bound HA as a ligand molecule to actively target cancer cells and to improve the intracellular uptake efficiency [38]. This is linked to overexpressed CD44 receptors on the surface of these cell lines [39], including the U87 glioblastoma cells [40]. Furthermore, owing to the hydrophilic and electronegative properties, the protein adsorption on nanoparticles will be impeded after HA modification [41,42]. By reducing the chance of macrophage recognition and to avoid the nonspecific cellular uptake, HA modification can effectively prolong the half-life of drug nanocarriers during circulation, and this results in better treatment outcomes [42,43].

Therefore, with the aim of enhancing the antitumor efficiency, we developed CDDP-loaded nanoparticles PMNPc by co-entrapping CDDP and IOMNP in poly(lactic-co-glycolic acid) (PLGA) with a single emulsion/evaporation step. The nanoparticle was surface modified through a layer-by-layer approach by chitosan and HA to prepare the HA/PMNPc, which can be used for dual-targeted drug delivery to cancer cells overexpressing the CD44 receptor on its surface. Furthermore, with a pH-sensitive drug release in the acidic endosomal environment after the intracellular uptake and NIR-inducible photothermal response from the IOMNP, the enhanced cytotoxicity toward U87 glioblastoma cells was demonstrated in vitro. Finally, the improved antitumor efficacy of HA/PMNPc was demonstrated in vivo for treating U87 xenografts in nude mice, for successful chemo-photothermal combination cancer therapy.

## 2. Materials and Methods

### 2.1. Materials

Iron (II) chloride tetrahydrate, iron (III) chloride hexahydrate, chitosan (CS, molecular weight = 50,000–190,000 Da, degree of deacetylation = 77%), cisplatin (CDDP), dichloromethane (DCM), dimethyl sulfoxide (DMSO), oleic acid (OA), and polyvinyl alcohol (PVA) (hydrolyzed, molecular weight = 30,000–70,000 Da) were supplied by Sigma-Aldrich (St Louis, MO, USA). Hyaluronic acid (HA) with 1,300,000 Da average molecular weight was provided by Bloomage Freda Biotechnology Co. (Jinan, China). Poly(lactic-co-glycolic acid) (PLGA) with 50% lactide and 50% glycolide and 15,000~30,000 Da molecular weight was supplied by Green Square Co. (Taipei, Taiwan). The U87 human primary glioblastoma cell line (ATCC HTB1) was obtained from American Type Culture Collection (Manassas, VA, USA) and genetically modified with firefly luciferase gene by lentiviral infection. The Dulbecco’s modified Eagle’s medium (DMEM) and fetal bovine serum (FBS) were obtained from Thermo Fisher Scientific (Waltham, MA, USA). The reagent for fluorescence staining, 4’,6-diamidino-2-phenylindole (DAPI), and phalloidin-tetramethylrhodamine B isothiocyanate (phalloidin-TRITC) were procured from Life Technologies (Carlsbad, CA, USA).

### 2.2. Preparation of PLGA Magnetic Nanoparticles (PMNP)

The iron oxide magnetic nanoparticles (IOMNP) were synthesized by the co-precipitation method. Briefly, 2.15 g FeCl_3_·6H_2_O and 0.79 g FeCl_2_·4H_2_O (Fe^3+^/Fe^2+^ molar ratio = 2/1) were dissolved in 50 mL deionized-distilled (DDI) water in a three-necked flat bottom flask. The flask was slowly heated to 60 °C after purging with nitrogen for 10 min under 500 rpm stirring. After slowly adding 5 mL 29.7% (*w*/*w*) NH_4_OH into the flask and reacting for 30 min under continuous stirring and nitrogen purging, the IOMNP black particles were washed three times with 50 mL DDI water with magnetic separation. The pH of the IOMNP solution was adjusted to 5 and sonicated for 10 min in an ice bath. The solution was purged with nitrogen for 10 min under 500 rpm stirring and heated to 60 °C, followed by adding 10 mL 30% (*w*/*w*) OA solution prepared in acetone, and reacted for additional 30 min. The OA-coated IOMNP (OMNP) was recovered and dispersed in chloroform to 10 mg/mL for storage at 4 °C.

The PLGA magnetic nanoparticles (PMNP) were synthesized by the emulsion/solvent evaporation method. Briefly, 50 mg PLGA was dissolved in 2.5 mL mixed organic solvent solution (2.25 mL acetone and 0.25 mL DCM) in a 4 mL vial, followed by adding 0.5 mL 10 mg/mL OMNP prepared above. The solution was vortexed for 1 min and sonicated for 1 min. This organic solution was added quickly into a 50 mL centrifuge tube containing 24 mL pH 7.4 phosphate-buffered saline (PBS) and vortexed, followed by sonication for 5 min. The mixed solution was added dropwise to a beaker filled with 50 mL PBS containing 0.3% (*w*/*v*) PVA and stirred at 1000 rpm and 35 °C for 12 h. The PMNP were recovered from the solution by magnetic separation, washed with PBS, and dried in a rotary evaporator under reduced pressure at 30 °C for 1 h. After washing three times with PBS, the PMNP were dispersed in DDI water for storage at room temperature.

### 2.3. Preparation of Hyaluronic Acid-Modified CDDP-Loaded PLGA Magnetic Nanoparticles (HA/PMNPc)

To prepare CDDP-loaded PLGA magnetic nanoparticles (PMNPc), the same procedure as in PMNP preparation was followed, except that 0.3 mL 20 mg/mL CDDP prepared in DMSO was added together with the OMNP solution. For preparation of fluorescence-labeled nanoparticles, the fluorescence dye was also added during this step. To modify the surface of PMNP with CS, 1 mg/mL PMNP in DDI water was added dropwise into 50 mL 2% (*w*/*v*) CS solution prepared in 1% (*v*/*v*) acetic acid and stirred at 300 rpm at room temperature for 1 h to prepare CS/PMNPc. After washing, the CS/PMNPc dispersed in DDI water was added dropwise into 50 mL 2% (*w*/*v*) HA solution prepared in DDI water and stirred at 300 rpm and room temperature for 1 h to prepare HA/PMNPc. The HA/PMNPc was washed 2 times and stored at room temperature in DDI water.

### 2.4. Drug Loading and Release

To determine the amount of CDDP entrapped in PMNPc, all supernatant during preparation of PMNPc was collected, for determination of the CDDP concentration by high-performance liquid chromatography (HPLC). After diluting with PBS, the sample was mixed with acetonitrile at 1:1 volume ratio, and 20 μL of the sample solution was injected into a JASCO HPLC system containing a PU-2089 pump and a UV-2075 detector for ultraviolet (UV) detection at 290 nm. The mobile phase was 0.01 M potassium dihydrogen phosphate:methanol:acetonitrile = 72:18:10 (*v*/*v*), and the flow rate was set at 0.5 mL/min. The amount of encapsulated CDDP was calculated from mass balance after subtracting the amount of un-encapsulated CDDP from the initial amount of CPPD added during preparation. The loading capacity (LC) and encapsulation efficiency (EE) of CDDP were calculated from the following equations.

$$\mathrm{Loading}\;\mathrm{capacity}\;\left(\mathrm{LC},\;\%\right)=\frac{\mathrm{Weight}\;\mathrm{of}\;\mathrm{encapsulated}\;\mathrm{CDDP}}{\mathrm{Weight}\;\mathrm{of}\;\mathrm{prepared}\;\mathrm{PMNPc}}\times 100\%$$

$$\mathrm{Encapsulation}\;\mathrm{efficiency}\;\left(\mathrm{EE},\;\%\right)=\frac{\mathrm{Weight}\;\mathrm{of}\;\mathrm{encapsulated}\;\mathrm{CDDP}}{\mathrm{Weight}\;\mathrm{of}\;\mathrm{added}\;\mathrm{CDDP}}\times 100\%$$

The pH-dependent drug release properties were analyzed by incubating PMNPc or HA/PMNPc in 10 mL PBS (pH 5 or 7.4). The solution was incubated at 37 °C in a shaking incubator at 50 rpm shaking speed. At specific times, all supernatant was collected by magnetic separation, followed by replenishing with 10 mL PBS of the same pH value as before. The CDDP concentration was determined by HPLC, and the cumulative drug release percentage was calculated based on the weight of CDDP in the nanoparticles for the drug release study.

### 2.5. Characterization of Nanoparticles

The particle morphology was analyzed using a transmission electron microscope (TEM, JEM-1230, JEOL, Tokyo, Japan) after diluting the sample to 0.1 mg/mL and dropping on a 200-mesh copper grid for drying in a 37 °C oven for 24 h. The particles size distribution and zeta potential were analyzed using a Zetasizer (Nano ZS 90, Malvern Instruments, Malvern UK). The thermal degradation behavior was determined from thermogravimetric analysis (TGA) using Q50 thermogravimetric analyzer (TA Instruments, New Castle, DE, USA) under nitrogen purging. All samples were dried and placed on a platinum pan. The temperature was raised from room temperature to 700 °C at 10 °C/min. The iron oxide (Fe_3_O_4_) content in nanoparticles was measured by digesting the sample in 37% (*w*/*v*) HCl at 60 °C for 2 h. The solution was diluted with DDI water and filtered with a 0.22 μm PVDF filter before measurement with an inductively coupled plasma optical emission spectrometer (ICP-OES, 710-ES, Varian, Palo Alto, CA, USA). An X-ray diffractometer (XRD, D2 Phaser, Bruker, MA, USA) was used to measure the crystalline structure of nanoparticles with copper Kα radiation at λ = 0.154060 nm and 2θ from 10° to 70° with 0.02° increment. From the XRD analysis, the magnetite crystalline size was calculated with the Debye–Scherrer equation from the highest diffraction peak. An MPMS-3 superconducting quantum interference device (SQUID, Quantum Design, San Diego, CA, USA) was used to measure the magnetization behavior of nanoparticles by determining the magnetic moment from −10,000 to 10,000 Oe at 298 °K.

### 2.6. In Vitro Cell Culture

#### 2.6.1. Intracellular Uptake

For all in vitro studies, U87 cells were cultured in DMEM supplemented with 10% FBS and 1% penicillin/streptomycin at 37 °C in a humidified 5% CO_2_ incubator. To evaluate the targeting efficiency after HA modification, U87 cells (2 × 10^4^ cells) were seeded in a 22 mm cover glass placed in a well of a 12-well cell culture plate and cultured for 24 h. The 5(6)-carboxyflurorescein (5(6)-FAM)-labeled PMNP or HA/PMNP (500 μg/mL in 0.1 mL DMEM medium) was added and incubated for another 24 h for intracellular uptake by U87 cells. The cells were washed with PBS and fixed with 10% (*w*/*v*) formaldehyde and incubated in 0.1% (*v*/*v*) Triton X-100 for 30 min. After washing with PBS, the sample was stained with 1 μg/mL phalloidin-TRITC for actin cytoskeleton and counterstained with 1 μg/mL DAPI for cell nucleus. The stained sample was observed under a confocal laser scanning microscopy (Zeiss LSM 510 Meta, Wetzlar, Germany) with the excitation wavelength at 350/492/577 nm (blue/green/red) and emission wavelength at 451/517/590 nm (blue/green/red). For intracellular uptake quantification by flow cytometry, U87 cells (2 × 10^5^) were cultured in a T-25 culture flask for 24 h, followed by adding 100 μL 5(6)-FAM-labeled PMNP or HA/PMNP (final particle concentration = 5 μg/mL) and incubated for another 24 h. After washing and detaching the cells with trypsin, a cell suspension was collected by a flow tube and analyzed by an Attune NxT flow cytometer (Thermo Fisher Scientific, Waltham, MA, USA).

#### 2.6.2. Cytotoxicity

The cytocompatibility of drug-free nanoparticles was first determined by adding 0.1 mL HA/PMNP (final particle concentration = 0.01 to 200 μg/mL) prepared in cell culture medium to U87 cells (2.5 × 10^3^) pre-cultured for 24 h in each well of a 96-well cell culture plate and cultured for another 24, 48, and 72 h before measuring the cell viability by the (3-(4,5-dimethylthiazol-2-yl)-2,5-diphenyl tetrazolium bromide) (MTT) assay. To carry out the MTT assay, the cell culture medium was removed, followed by adding 100 μL 0.5 mg/mL MTT solution and incubated for 2 h at 37 °C in a CO_2_ incubator. By dissolving the water-insoluble formazan crystal salt produced by the metabolically active cells in DMSO, a microplate reader (Synergy HT, BioTek, Winooski, VT, USA) was used to measure the absorbance at 570 nm, which is in proportion to the cell viability. To evaluate cytotoxicity of nanoparticles, U87 cells (5 × 10^3^ cells) were seeded in each well of a 96-well cell culture plate and pre-cultured for 24 h. After removing the cell culture medium and replenishing with 0.1 mL CPPD, PMNPc, or HA/PMNPc solution (in cell culture medium), which contain different concentrations of free or encapsulated CDDP. The cell culture was continued for another 72 h at 37 °C in a CO_2_ incubator, and cell viability was determined by MTT assays as described before. The drug concentrations achieving 50% cytotoxicity (IC50) were calculated using a four-parameter logistic function.

#### 2.6.3. Apoptosis

To evaluate cell apoptosis, flow cytometry analysis and Western blot analysis were used. For chemo treatment, 2 × 10^5^ U87 cells were cultured in T-25 flask for 24 h and treated with PMNP, PMNPc, 0.5 μg/mL CDDP, or HA/PMNPc (drug dosage = 0.5 μg/mL CDDP) for 72 h before collecting into a 2 mL centrifuge tube. For photothermal treatment, U87 cells (1 × 10^6^ cells in 0.2 mL medium) were collected into a 2 mL centrifuge tube, incubated with 200 μL PBS or HA/PMNP (final particle concentration = 5 μg/mL) for 2 h, and treated with 808 nm NIR laser at 2 W/cm^2^ for 2 min. For flow cytometry analysis, the centrifuge tube was centrifuged for 5 min, followed by removing the supernatant and adding 0.5 mL binding buffer to disperse the cells. After adding 5 μL Annexin V-FITC and 5 μL Propidium iodide (PI) into the tube and incubated for 5 min, the sample was analyzed by an Attune NxT flow cytometer (Thermo Fisher Scientific, Waltham, MA, USA). For Western blot analysis, the centrifuge tube was centrifuged for 5 min, followed by removing the supernatant and adding 0.5 mL RIPA lysis buffer to lyse the cells. The standard protocol of Western blot was used with rabbit anti-mouse primary antibodies for β-actin (4970L, Cell Signaling, 1:1000), extracellular-signal-regulated kinase (ERK) (MA5-15134, Invitrogen, 1:1000), phosphorylated extracellular-signal-regulated kinase (phospho-ERK) (4376S, Cell Signaling, 1:1000), caspase-3 (GTX110543, GeneTex, 1:1000), and cleaved caspase-3 (9661S, Cell Signaling, 1:1000). After incubating with a goat anti-rabbit secondary antibody (7074S, Cell Signaling, 1:1000) and HRP substrate (Millipore WBKLS0500), images were taken using a gel imaging system (MultiGel-21, TopBio, New Taipei City, Taiwan).

### 2.7. In Vivo Study

#### 2.7.1. Xenograft Tumor Models

For in vivo experiments, all procedures were performed as per protocols approved by the Institutional Animal Care and Use Committee of Chang Gung University with CGU108-214 IACUC approval number and 16 April 2020 approval date. The U87 tumor-bearing xenograft model was obtained by subcutaneous injection of 5 × 10^7^ U87 cells to the right flank of 4-week-old female BALB/c nude mice. To perform the magnetic targeting study, 100 μL of PBS or Cy5.5-labeled HA/PMNPc (7.5 mg/mL) was injected via the tail vein six days after injecting the tumor cells (n = 1). A 2400 Gauss magnet (10 × 5 × 2 mm) was used for magnetic guidance for 1 h around the tumor region in the HA/PMNPc (M+) group vs. the non-targeted HA/PMNPc (M−) group. The mice were sacrificed 4 h after administration of sample, and major organs and tumor tissue were explanted to compare the extent of nanoparticle accumulation in the organs and tumors with an in vivo imaging system (IVIS) (Lumina LT, Perkin-Elmer, Waltham, MA, USA). The mice in the HA/PMNPc (M+) group were also separately subject to 808 nm NIR laser irradiation at the tumor region using different laser power (1.5, 1.75, or 2 W/cm^2^) and irradiation times (1, 2, 3, 4, or 5 min), and time-lapsed tumor temperatures were monitored with an infrared camera for up to 5 min.

For anticancer efficacy study, the mice were randomly divided into 5 groups seven days after subcutaneous implantation of U87 cells, and a 100 μL sample was administrated intravenously for treatment. The control group is injection of PBS; the CDDP group is injection of CDDP at 1 mg/kg drug dosage; the HA/PMNPc (M−/L−) is injection of HA/PMNPc at 1 mg/kg drug dosage; the HA/PMNPc (M+/L−) is injection of HA/PMNPc at 1 mg/kg drug dosage with magnetic field guidance; the HA/PMNPc (M+/L+) group is injection of HA/PMNPc at 1 mg/kg drug dosage with magnetic field guidance and NIR laser irradiation. The treatment was carried out on days 8, 12, 15, and 19. For magnetic field guidance (M+), a 2400 Gauss magnet (10 × 5 × 2 mm) was placed near the tumor for 1 h. For laser irradiation (L+), the magnet was removed, and the tumor was exposed to 808 nm NIR laser at 1.75 W/cm^2^ for 5 min. The body weight was recorded, and the tumor volume was determined by measuring the width and length of the tumor with a caliper. Mice were sacrificed when the tumor volume exceeded 1000 mm^3^, from which the survival time of the animal was determined. The bioluminescence intensity (BLI) of tumor was determined from IVIS after injection of 100 μL 15 mg/mL luciferin solution. The tumor volume and normalized BLI is calculated as follows.

$$\mathrm{Tumor}\;\mathrm{volume}=\frac{\mathrm{Length}\;\times \;\mathrm{width}\;\times \;\mathrm{width}}{2}$$

$$\mathrm{Normalized}\;\mathrm{BLI}=\frac{\mathrm{BLI}\;\mathrm{intesnity}\;\mathrm{at}\;a\;\mathrm{specific}\;\mathrm{time}}{\mathrm{BLI}\;\mathrm{intensity}\;\mathrm{at}\;\mathrm{day}\;7}$$

#### 2.7.2. Histological Analysis

For histological analysis, tumor tissues were collected after sacrificing the mice and fixed in a 10% (*w*/*v*) formaldehyde solution. The tissues were sectioned into 5 μm thickness on a glass slide after paraffin embedment. Slides were then subject to hematoxylin and eosin (H&E) and immunohistochemical (IHC) staining following standard protocols [40]. For IHC staining, rabbit anti-mouse phospho-ERK (4376S, Cell Signaling, 1:200) or cleaved caspase-3 primary antibody (9661S, Cell Signaling, 1:100) was used. After incubating with a secondary antibody (HRP Polymer Quanto, TL-060-QPB, Thermo Fisher Scientific) and hematoxylin, images were taken using an inverted microscope (IX71, Olympus, Tokyo, Japan).

### 2.8. Statistical Analysis

Data are presented as mean ± standard deviation (SD). Statistical analyses were performed using one-way analysis of variance (ANOVA) analysis and *p* values less than 0.05 indicate statistical significance.

## 3. Results and Discussion

### 3.1. Preparation and Characterization of Nanoparticles

**Figure 1:**
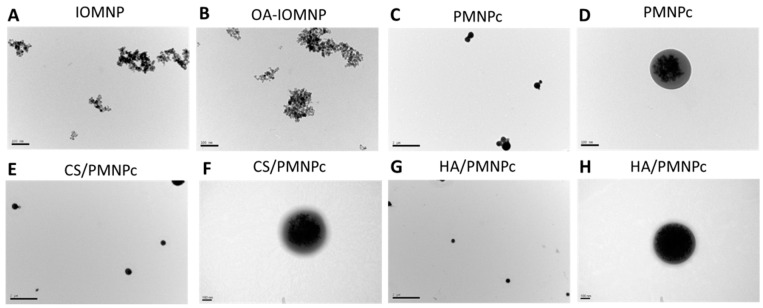
The transmission electron microscope (TEM) images of iron oxide magnetic nanoparticles (IOMNP) ((**A**) bar = 100 nm), oleic acid (OA)-coated IOMNP (OA-IOMNP) ((**B**) bar = 100 nm), CDDP-loaded PLGA magnetic nanoparticles (PMNPc) ((**C**) bar = 2 μm; (**D**) bar = 100 nm), chitosan (CS)-coated PMNP (CS/PMNPc) ((**E**) bar = 2 μm; (**F**) bar = 100 nm), and HA-coated PMNPc (HA/PMNPc) ((**G**) bar = 2 μm; (**H**) bar = 100 nm).

The nanoparticles were synthesized via a single oil-in-water emulsion by entrapping the oleic acid (OA)-coated IOMNP (OA-IOMNP). To obtain nanoparticles of a uniform size and surface morphology, the preparation condition during the emulsification step was studied and optimized to achieve the best preparation conditions for the PMNP. Under the same preparation condition, cisplatin (CDDP) was added during the emulsification step to fabricate the PMNPc, and this nanoparticle was further successively surface modified with CS and HA to prepare the HA/PMNPc. To disperse the IOMNP in the solution, the OA-coated IOMNP were used for better distribution to the organic phase during the emulsification step. The IOMNP and OA-IOMNP tend to agglomerate in water as shown from the transmission electron microscope (TEM) images, where a small aggregate was observed with a discrete nanoparticle of 10~20 nm in size (Figure 1A,B). This aggregation behavior mainly arises from the Van der Waals force or a weak interaction from the magnetic dipole–dipole force [44], which was observed before for IOMNP prepared by a similar co-precipitation method as this study [45,46]. After co-encapsulating the CDDP and OA-IOMNP in the PLGA, the PMNPc showed less aggregation (Figure 1C), with a spherical and smooth particulate morphology and infused with a black core of agglomerated nanoparticles (Figure 1D). This indicates the OA-IOMNP could be well encapsulated within the polymeric matrix during the emulsion/solvent evaporation process. A sequential surface coating with CS and HA apparently causes little change in the surface morphology of the nanoparticles, judging from the TEM images of the CS/PMNPc (Figure 1E,F) and HA/PMNPc (Figure 1G,H), which does not show a substantial change in the particle morphology from the PMNPc. Nonetheless, a light gray layer appears on the surface of these particles, possibly due to the deposition of CS and HA on the PMNPc surface during the layer-by-layer coating step.

**Figure 2:**
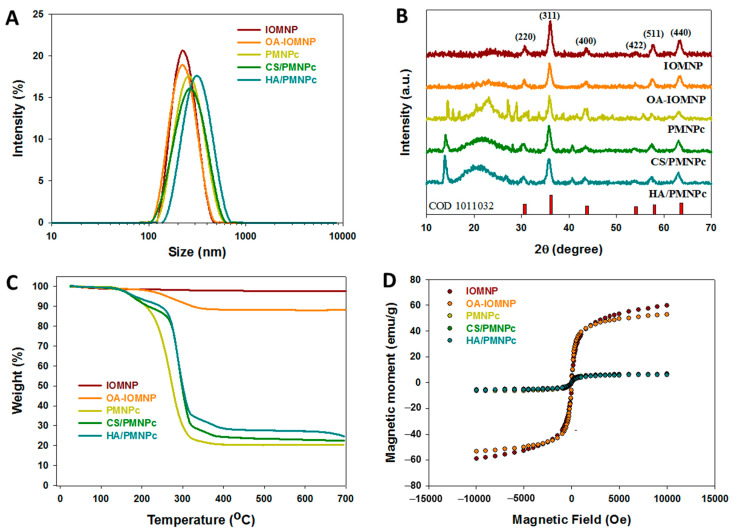
The dynamic light scattering (DLS) (**A**), X-ray diffraction (XRD) analysis (**B**), thermogravimetric analysis (TGA) (**C**), and superconducting quantum interference device (SQUID) analysis (**D**) of iron oxide magnetic nanoparticles (IOMNP), oleic acid (OA)-coated IOMNP (OA-IOMNP), CDDP-loaded PLGA magnetic nanoparticles (PMNPc), chitosan (CS)-coated PMNPc (CS/PMNPc), and HA-coated PMNPc (HA/PMNPc).

From the dynamic light scattering (DLS) analysis, a single distribution peak was found (Figure 2A). The mean values of the average hydrodynamic diameters of the IOMNP, OA-IOMNP, PMNPc, CS/PMNPc, and HA/PMNPc are 218.0, 230.7, 242.2, 277.2, and 337.4 nm, respectively (Table 1). Indeed, the particle size is in the order of PMNPc < CS/PMNPc < HA/PMNPc as the CS and HA are sequentially coated on the particle surface. The polydispersity index (PDI) values for all were below 0.30 (Table 1), indicating these nanoparticles have good colloidal stability [47]. The average zeta potential from the electrophoretic mobility measurements shows a positive value (22.1 mV) for the IOMNP due to the residual ammonium ions, as ammonia was used for the co-precipitation of the ferric and ferrous ions (Table 1). However, due to the presence of the carboxylate group in the OA and PLGA, the zeta potential changed to a negative value for the OA-IOMNP (−18.4 mV) and PMNPc (−25.2 mV). The surface potential changed dramatically to 28.4 mV after coating the PMNPc with the cationic polymer CS, and the value further shifted to a negative one (−30.9 mV) after modifying with the anionic polymer HA as expected.

The X-ray diffraction (XRD) patterns shown in Figure 2B indicate six characteristic peaks for all the samples, representing the (220), (311), (400), (422), (511), and (440) lattice planes of the magnetite structure (COD 1011032) [48,49]. This also confirms that iron oxide nanoparticles after an OA coating or PLGA encapsulation will not change their spinel structure. The average crystal size calculated from the Scherrer equation with XRD line broadening, assuming spherical crystals and using the highest diffraction peak, ranges from 9.8 to 10.6 nm (Table 1). From the TGA results, the final residual weight at 700 °C is 97.7 and 88.3% for the IOMNP and OA-IOMNP with weight loss from the decomposition of the OA (Figure 2C). For the PLGA-based nanoparticles, a substantial weight loss near 275 °C was evident due to the PLGA decomposition, and the final residual weight of the PMNPc, CS/PMNPc, and HA/PMNPc was 20.5, 22.4, and 24.6%, respectively (Figure 2C). The residual weight is in the order HA/PMNPc > CS/PMNPc > PMNPc, indicating the successful modification of the PMNPc with CS and HA as the residual weight of a nanoparticle after a complete thermal decomposition in nitrogen will increase with the presence of CS or HA on the particle surface with some residual weight shown by those natural polymers. This is different from a zero residual weight shown for a synthetic polymer like the PLGA [50].

The magnetization curves at room temperature from the superconducting quantum interference device (SQUID) analysis are shown in Figure 2D. The hysteresis loops passed through the origin of the axes, and the remnant (residual) magnetization without the magnetic field was close to zero, indicating the superparamagnetic behavior for all the tested samples [51,52]. As the superparamagnetism of the magnetic nanoparticles depends on the size of the IOMNP, which occurs when the particle size is below ~20 nm, this behavior is consistent with the particle size determined from the TEM and XRD. One advantage of this superparamagnetic property is that the nanoparticles could be magnetically guided to the tumor site to exert their action and be dispersed easily thereafter by removing the magnetic field without creating residual magnetic interactions between the nanoparticles to form aggregates, which can prevent a possible complication after treatment. The saturation magnetization values are 59.6 and 52.8 emu/g for the IOMNP and OA-IOMNP, respectively. These values decreased to 6.5, 6.3, and 5.9 emu/g for the PMNPc, CS/PMNPc, and HA/PMNPc, respectively, due to the reduced IOMNP weight percentage in the nanoparticles. Although these saturation magnetization values are sufficient for the magnetic guidance during the magnetically targeted drug delivery, dual targeting by a combination with a CD44-targeting HA ligand will undoubtedly improve the targeting efficacy to enhance the drug delivery efficiency [53]. To confirm that the reduced saturation magnetization value was due to the non-magnetic component PLGA, the Fe_3_O_4_ content in each sample was estimated by dividing the saturation magnetization value of each sample by the value of the IOMNP (59.6 emu/g) and assuming the IOMNP to be 100% Fe_3_O_4_ (Table 1). Alternatively, the Fe_3_O_4_ contents measured directly from the inductively coupled plasma optical emission spectrometry (ICP-OES) are also shown in Table 1, which are comparable with those calculated from SQUID. Overall, this confirms the reduced saturation magnetization value originated from the change in the IOMNP content in the nanoparticles, and the HA/PMNPc nanoparticle contains ~10% (*w*/*w*) IOMNP.

The Fourier transform infrared (FTIR) spectroscopy analysis of all the synthesized nanoparticles is shown in Figure S1 (Supplementary Materials). By comparing with the FTIR spectra of all the components in the nanoparticles, we could confirm the successful synthesis of the HA/PMNPc. The photothermal response of the HA/PMNPc is shown in Figure S2 (Supplementary Materials). The temperature of an HA/PMNPc aqueous solution could reach ~50 °C after irradiating with an 808 nm NIR laser for 3 min, endorsing its use for cancer photothermal therapy. The photothermal conversion efficiency was calculated from a heating–cooling cycle during laser treatment and the value is 25.53% (Figure S3, Supplementary Materials). To reveal the post-photothermal stability, the temperature profile during five repeated heating–cooling cycles after the laser treatment is shown in Figure S4 (Supplementary Materials). The temperatures after the 180 s laser treatment time are 52.2, 52.0, 52.2, 51.8, and 51.8 °C in each cycle, indicating the excellent photothermal stability of the HA/PMNPc when repeatedly irradiated with an 808 m laser light.

**Figure 3:**
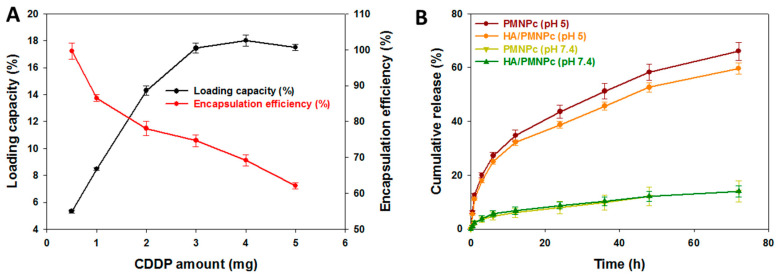
(**A**) The loading capacity (LC) and encapsulation efficiency (EE) of CDDP in PMNPc. (**B**) The release profiles of CDDP from CDDP-loaded PLGA magnetic nanoparticles (PMNPc) and HA-coated PMNPc (HA/PMNPc) at 37 °C in pH 5 and pH 7.4 phosphate-buffered saline (PBS).

The loading capacity (LC, the weight percentage of the drug in the PMNPc) and the encapsulation efficiency (EE, the weight percentage of the initially added drug encapsulated in the PMNPc) of the CDDP are shown in Figure 3A. By increasing the amount of CDDP used for preparing the PMNPc from 0.5 to 5 mg, the LC value increases accordingly from 5.3% to reach a maximum value (~18.0%) when 3 mg CDDP was used in the formulation. However, the EE value decreases from 99.6 to 62.1% with an increasing CDDP amount. As the LC could not increase further, but the EE decreases, a 3 mg CDDP dosage was chosen for preparing the PMNPc in all the studies. The release of CPPD from the PMNPc and HA/PMNPc at 37 °C in a pH 5 or 7.4 PBS is shown in Figure 3B. The drug release exhibits a pH sensitivity for both the PMNPc and HA/PMNPc, but a faster drug release rate is observed at pH 5. Indeed, the cumulative release percentages in 72 h for the PMNPc are 11.4 and 63.8% at pH 7.4 and pH 5, respectively, which are 11.5 and 57.8% for the HA/PMNPc. The burst release at pH 5 during the initial stage (to 8 h) may be due to the weakened coordination bonding between the CPPD and PLGA in the acidic solution [54]. Alternatively, a reduced electrostatic interaction or hydrogen bonding between –NH_3_ in CDDP and –COOH/–OH in PLGA from H^+^ may also exist [55,56]. At the later stage (from 8 to 72 h), the drug release rate at pH 5 is still higher than at pH 7.4, which may be owing to the fast degradation of the PLGA in an acidic environment, which induces the sustained release of the CDDP [57]. An acidic environment (~pH 5) resembles the endosomal microenvironment after endocytosis of the HA/PMNPc by tumor cells while the extracellular environment is at a physiological pH value of 7.4. Therefore, the release of the CDDP from the HA/PMNPc will be enhanced in the endosomes after the intracellular uptake to exert a higher cytotoxicity toward cancer cells for chemotherapy.

### 3.2. In Vitro Studies

**Figure 4:**
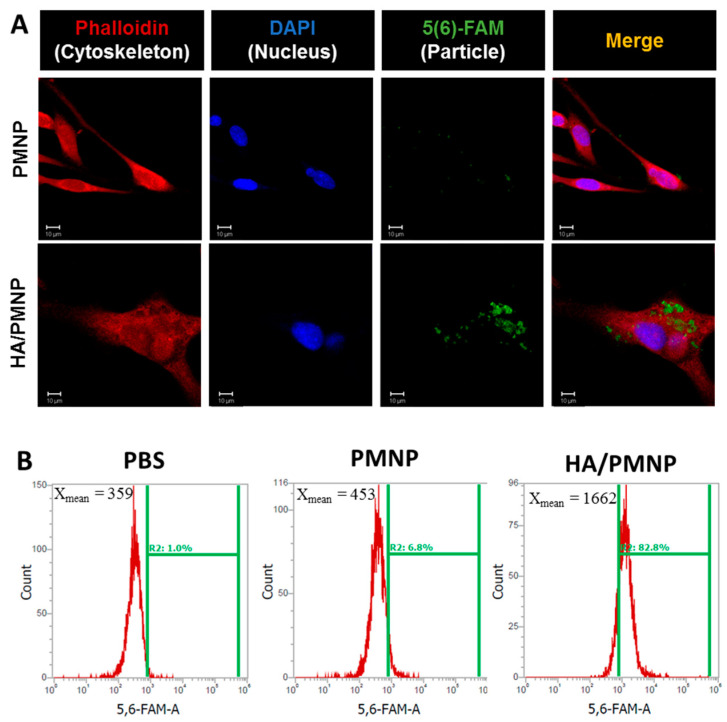
The in vitro targeting efficiency from confocal microscopy ((**A**) bar = 10 μm) and flow cytometry (**B**) analysis after incubating U87 cells with 5(6)-carboxyfluorescein (5(6)-FAM)-labeled PLGA magnetic nanoparticles (PMNP) and (5(6)-FAM)-labeled HA-coated PLGA magnetic nanoparticles (HA/PMNP) for 24 h. The cytoskeleton was labeled with phalloidin-TRITC (red) and the nucleus was counterstained with DAPI (blue).

The intracellular uptake efficiency was studied by incubating U87 cells with fluorescein-labeled PMNP and HA/PMNP for 24 h to evaluate the utility of HA as a CD44-targeting ligand. The 5(6)-carboxyfluorescein (5(6)-FAM) was added during the emulsion step of the particle preparation to encapsulate this fluorescence dye within the nanoparticles, which can be tracked from the emitted intracellular green fluorescence signal when examined under a confocal laser scanning microscope. For the visualization of the intracellular uptake, the cell cytoskeleton was stained with red fluorescence-producing phalloidin-TRITC and the nucleus labeled with blue fluorescence-producing DAPI. As shown in Figure 4A, the green fluorescence intensity associated with the nanoparticle is much stronger for the HA/PMNP than the PMNP, indicating an HA surface coating will increase the efficiency of the nanoparticle engulfment by U87 through CD44-mediated endocytosis [58,59]. The intracellular uptake efficiency was further subject to a quantitative analysis using flow cytometry by determining the intracellular fluorescence signal intensity associated with the fluorescein-labeled nanoparticles with fluorescence-activated cell sorting analysis. As shown in Figure 4B, the geometric mean fluorescence intensities are 453 and 1662 and the nanoparticle uptake ratio is 6.8 and 82.8% for the PMNP and HA/PMNP, respectively. After subtracting the background signal from the PBS control, the HA/PMNP is endowed with a ~13 times higher targeting efficacy than the PMNP due to the enhanced cellular trafficking mediated by the ligand–receptor interaction, which is in line with the trends observed from the confocal microscopy analysis (Figure 4A). Overall, the intracellular uptake study endorses the preferred use of HA/PMNP for drug delivery to U87 glioblastoma cells.

**Figure 5:**
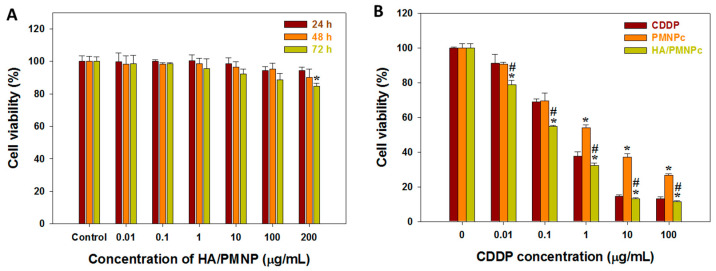
(**A**) The cytocompatibility of HA/PMNP by incubating different concentrations of nanoparticles with U87 cells for 24, 48, and 72 h and determining the cell viability by MTT assays. The control is cell culture medium. * *p* < 0.05 compared with 24 h. (**B**) The cytotoxicity of CDDP, CDDP-loaded PLGA magnetic nanoparticles (PMNPc), and HA-modified PMNPc (HA/PMNPc) after incubating with U87 cells for 72 h and determining the cell viability by MTT assays. * *p* < 0.05 compared with CDDP, ^#^ *p* < 0.05 compared with PMNPc.

As shown in Figure 5A, the drug-free nanoparticle HA/PMNP elicits negligible cytotoxicity toward U87 from 0.01 to 200 μg/mL particle concentrations. Indeed, at the highest HA/PMNP concentration, the cell survival rate is still more than 85%, underlining the high cytocompatibility of a drug-free HA/PMNP nanocarrier. After confirming the non-toxic nature of the nanocarrier, the cytotoxicity of a drug-loaded nanocarrier (HA/PMNPc) was next studied in vitro against U87 cancer cells. The cell viability was compared with those from the free CDDP and non-targeted drug-loaded nanocarrier (PMNPc) from 0.01 to 100 μg/mL drug concentrations. As shown in Figure 5B, the cytotoxicity increases with the CDDP concentration as expected, where the drug concentrations achieving 50% cytotoxicity (IC50) are 0.648, 3.180, and 0.297 μg/mL for the CDDP, PMNPc, and HA/PMNPc, respectively. Compared with the CDDP, the PMNPc shows a lower cytotoxicity and an increased IC50 value, mainly arising from the passive accumulation of the negative-charged PMNPc around the U87 cells. The inefficient intracellular uptake rate as well as the low extracellular drug release rate at pH 7.4 results in a 4.9-fold increase in the IC50 of the PMNPc over the free drug. In contrast, by the active targeting to the U87 to enhance the intracellular uptake rate as well as a much higher drug release rate in the acidic environments of endosomes, the HA/PMNPc reveals an enhanced cytotoxicity and the IC50 value is only 46% that of the free drug.

**Figure 6:**
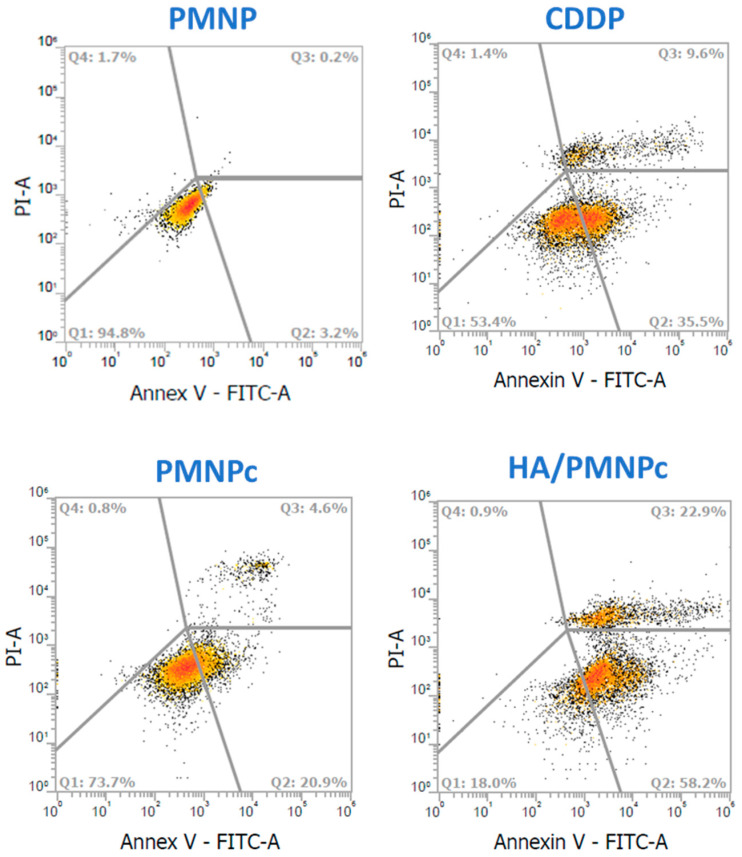
The cytotoxicity of CDDP, CDDP-loaded PLGA magnetic nanoparticles (PMNPc), and HA-coated PMNPc (HA/PMNPc) after incubating with U87 cells for 72 h. The cell apoptosis/necrosis was determined by flow cytometry analysis using Annexin V-FITC/PI staining. Q1: live; Q2: early apoptosis; Q3: late apoptosis; Q4: necrosis. The tested drug concentration is 0.5 μg/mL.

The cell death mechanism induced by CDDP was investigated by a flow cytometry analysis and is shown in Figure 6, where Q1 is the percentage of live cells, Q2 is the early apoptosis, Q3 is the late apoptosis, and Q4 is necrosis. The cell survival rate after the drug-free PMNP treatment is 94.8% due to its non-toxic nature and high cytocompatibility, as shown in Figure 5A. For CDDP-induced cytotoxicity, the cell death mechanism is mainly due to apoptosis rather than necrosis, supporting the primary cytotoxic mechanism of CDDP by DNA binding to induce DNA damage and subsequently lead to cell apoptosis [60]. The total apoptosis rates (early + late) are 45.1, 25.5, and 81.1% for the CDDP, PMNPc, and HA/PMNPc, respectively, which is consistent with the trend of the IC50 values observed before. Taken together, the minimum percentage of live cells in the HA/PMNPc group (18.0%) endorses the use of HA/PMNPc for targeted chemotherapy in CDDP delivery.

**Figure 7:**
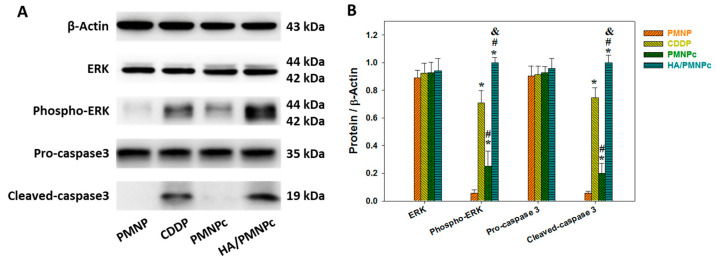
(**A**) The expression of apoptosis marker proteins by U87 cells from Western blot analysis after incubating with CDDP, CDDP-loaded PLGA magnetic nanoparticles (PMNPc), and HA-coated PMNPc (HA/PMNPc) for 72 h. (**B**) The semi-quantitative analysis of Western blot results. The tested drug concentration is 0.5 μg/mL. * *p* < 0.05 compared with PMNPc; ^#^ *p* < 0.05 compared with CDDP; ^&^ *p* < 0.05 compared with HA/PMNPc.

As cell apoptosis involves the cleavage of the pro-caspase, the protein expression of the cleaved caspase-3 could be used as a reliable indicator for cell apoptosis [61]. By increasing the phosphorylation level and activation of the extracellular-signal-regulated kinase (ERK) protein, the synthesis of the protein phosphorylated-ERK (phospho-ERK) is another signaling molecular pathway of cell apoptosis [62]. We therefore also used the Western blot to analyze the expression of these apoptosis marker proteins. From the Western blot results, the U87 cells treated with PMNP show the minimum production of the cleaved caspase-3 and phospho-ERK, echoing its high biocompatibility as shown before (Figure 7A). Furthermore, as there is no significant difference in the protein expression level of the ERK and pro-caspase-3 among all the groups, but only the CDDP-treated groups show an increased production of phospho-ERK and cleaved caspase-3 apoptosis marker proteins, the validity of using the Western blot for the apoptosis analysis is confirmed. The anticancer mechanism of the CDDP is by drug binding to the DNA and destroying the DNA helical structure to inhibit the translation inhibition and induce cell apoptosis [63]. Furthermore, the drug also generates reactive oxygen species in a variety of ways, which can promote ERK phosphorylation and activate and cleave pro-caspase-3 during cell apoptosis [64]. Using a semi-quantitative analysis of the Western blot results, the CDDP-treated groups showed a significant difference in the cleaved caspase-3 and phospho-ERK protein production from the drug-free PMNP and among themselves, in the order of HA/PMNPc > CDDP > PMNPc. This trend is in line with the results from the previous cytotoxicity analysis and supports that the drug mechanism of CDDP is by causing cell apoptosis (Figure 7B).

**Figure 8:**
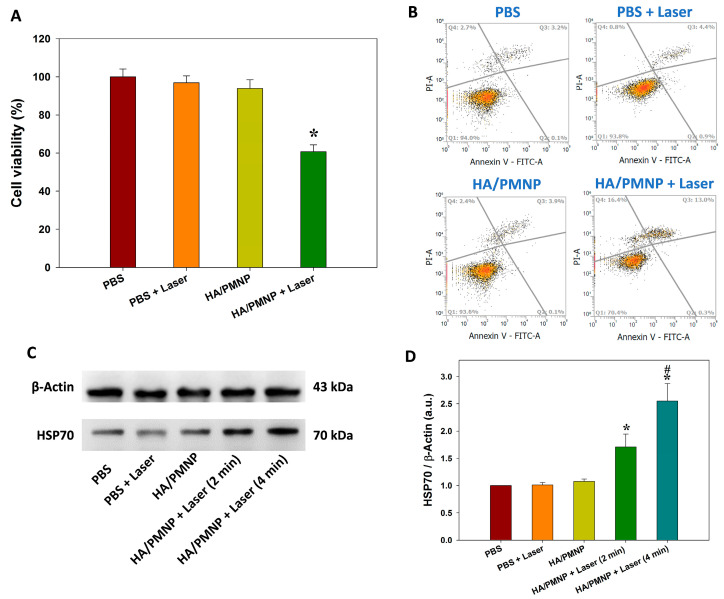
The cytotoxicity after treating U87 cells with PBS, or HA-coated PLGA magnetic nanoparticles (HA/PMNP) in PBS for 24 h followed by 880 nm near-infrared (NIR) laser irradiation. (**A**) The cell viability from MTT assays after 2 min laser irradiation. * *p* < 0.05 compared with HA/PMNP. (**B**) The quantification of cell apoptosis and necrosis by flow cytometry after 2 min laser irradiation with Annexin V-FITC/PI staining. Q1: live; Q2: early apoptosis; Q3: late apoptosis; Q4: necrosis. (**C**) The Western blot analysis of heat-shock protein 70 (HSP70). (**D**) The semi-quantitative analysis of production of HSP70 from Western blot. * *p* < 0.05 compared with HA/PMNP; ^#^ *p* < 0.05 compared with HA/PMNP + Laser (2 min).

The cytotoxicity toward the U87 from the HA/PMNP-mediated photothermal treatment was studied next. As shown in Figure 8A, when the PBS was used to treat the U87 cells, the NIR laser irradiation for 2 min (PBS + Laser) does not lead to any cell death. In contrast, a significant reduction in the cell viability (33%) was found when the U87 cells were treated with HA/PMNP and followed by NIR laser irradiation for 2 min (HA/PMNP + Laser), in contrast to an insignificant cell viability change from the PBS without NIR laser irradiation (HA/PMNP). As the IOMNP encapsulated in HA/PMNP can generate heat when exposed to NIR light, the HA/PMNP can be considered as an efficient photothermal agent for photothermal treatment with its high intracellular uptake rate by U87 cells [65]. The mechanism of the NIR-induced cell death was further confirmed from the flow cytometry. As shown in Figure 8B, the PBS group shows a minimum change in the apoptosis/necrosis rate after the laser irradiation. However, a vast increase in the late apoptosis/necrosis rate from 6.3 to 29.4% was noted in the HA/PMNP group, revealing the manifested cell apoptosis/necrosis after the photothermal treatment [66]. Overall, this supports the NIR-responsive nature of HA/PMNP by inducing cell death from its photothermal conversion ability [67]. The tumor cells commonly overexpress heat-shock proteins to prevent heat damage [68]. These protein are stress inducible and are frequently used as cellular markers when the cells are experiencing a thermal stress [69]. Specifically, heat-shock protein 70 (HSP70) is a group of protective proteins whose production could be triggered from a temperature rise, rendering its use as an indicator during photothermal treatment [70,71]. Therefore, we also use Western blot to analyze the HSP70 production by U87 as an indicator of the photothermal response from the HA/PMNP. As shown in Figure 8C, the PBS, PBS + Laser, and HA/PMNP groups reveal the background production of the constitutive HSP70 protein by U87 cells. The semi-quantitative analysis of the Western blot results also shows no significant change in the HSP70 production between these groups (Figure 8D). The HSP70 production was apparently increased by irradiating with the NIR laser for 2 min, which could be further upregulated with longer laser irradiation time up to 4 min. Therefore, the upregulation of the HSP70 expression is correlated with the laser exposure time and the thermal stress experienced by the cells. A significant increase in the HSP70 production by U87 is found in 4 min compared with no laser treatment or 2 min laser irradiation time (Figure 8D). Specifically, the exposure of the HA/PMNP-treated U87 cells to an NIR laser for 2 min results in a 1.7-fold increase over the group without laser light exposure, which could be further increased to 2.6-fold by extending the laser exposure time to 4 min. This time-dependent upregulation of the HSP70 protein production undoubtedly confirms the mechanism of the NIR-induced photothermal treatment with HA/PMNP.

### 3.3. In Vivo Studies

**Figure 9:**
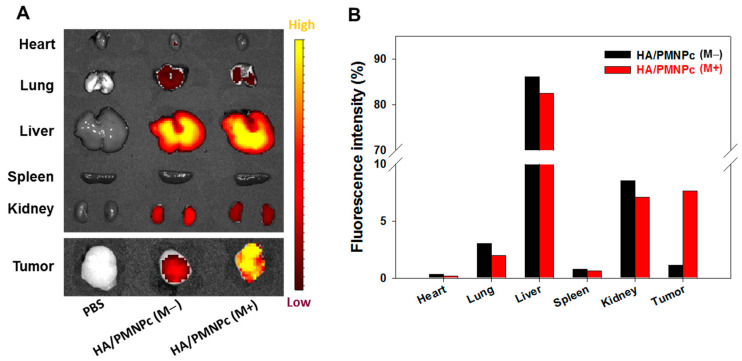
The biodistribution of Cy5.5-labeled HA/PMNPc in U87 tumor-bearing nude mice was determined by injecting 100 μL PBS or HA/PMNPc nanoparticles through the tail vein with (M+) or without (M−) magnetic targeting, using a magnet at the tumor area (n = 1). The in vivo imaging system (IVIS) was used for ex vivo imaging of explanted organs and tumors after 4 h (**A**), and quantification of distribution of nanoparticles was calculated based on fluorescence intensity in each organ as well as tumor (**B**).

The in vivo magnetic targeting ability of HA/PMNPc was first studied using the IVIS fluorescence imaging of retrieved major organs and explanted tumors 4 h after intravenous injection of PBS or Cy5.5-labeled HA/PMNPc. A 2400 Gauss magnet was used for magnetic guidance around the tumor region in the HA/PMNPc (M+) group. As shown from Figure 9A, the control PBS group did not reveal any fluorescence signal, in contrast to the HA/PMNPc groups. Using the percentage of fluorescence intensity found in the major organs and tumors after sacrificing the animals, most of the nanoparticles were found to be accumulated in the liver, regardless of magnetic guidance or not (Figure 9B) [72]. This limited tumor delivery efficacy is, in general, in line with the results from previous comprehensive biodistribution studies in anticancer nanomedicine [73,74]. Nonetheless, when the magnetic guidance was introduced by placing a magnet around the subcutaneously implanted tumor region after the intravenous administration of HA/PMNPc, a substantial increase in the fluorescence intensity was found from the explanted tumors with ex vivo fluorescence imaging (Figure 9A). Indeed, a 6.8-fold increase in the tumor-targeting efficacy could be achieved in the HA/PMNPc (M+) group, when a magnet was used for the magnetic guidance after administrating the HA/PMNPc, compared with the HA/PMNPc (M−) group that does not use a magnet. Taken together, this supports the use a dual targeting strategy for treating xenograft tumors in nude mice.

**Figure 10:**
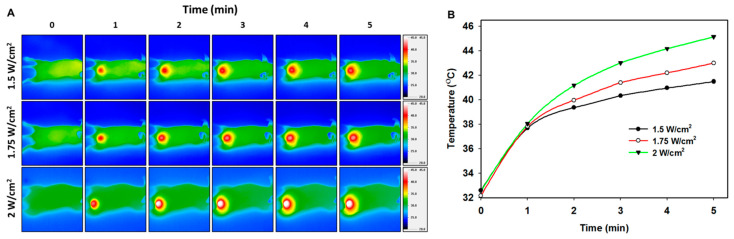
(**A**) The thermal images using an infrared camera, and (**B**) the time-dependent peak temperature profiles in the tumor area of U87 tumor-bearing nude mice after 808 nm NIR laser irradiation. The HA/PMNPc solution (100 μL) was used for intravenous injection, followed by magnetic targeting with a magnet and NIR laser irradiation at the tumor area.

The in vivo photothermal effect was studied next to judge the applicability of combining photothermal therapy with chemotherapy in vivo. The mice in the HA/PMNPc (M+) group were subjected to NIR laser irradiation at the tumor region with different laser powers, and a time-lapsed tumor temperature profile was recorded with an infrared camera up to 5 min (Figure 10A). As shown in Figure 10B, the peak tumor temperature increases at a higher laser power, and the peak tumor temperature could reach ~41, ~43, and ~45 °C in 5 min. During cancer photothermal therapy, the tumor temperature can be raised above 50 °C by using a higher laser density or a prolonged laser irradiation time to induce the necrosis of the tumor cells [75]. Nevertheless, this kind of harsh condition may lead to a nonspecific injury to normal tissues, vasculature, and host antitumor immunity [76]. A mild-temperature photothermal therapy for tumor ablation is therefore more desirable, with a temperature higher than the physiological temperature (> 42 °C) but less than 45 °C, to circumvent the issues associated with high-temperature photothermal therapy but still preserving the therapeutic efficacy. Considering this, we chose 1.75 W/cm^2^ laser power with 5 min irradiation time for treating U87 tumor-bearing nude mice.

**Figure 11:**
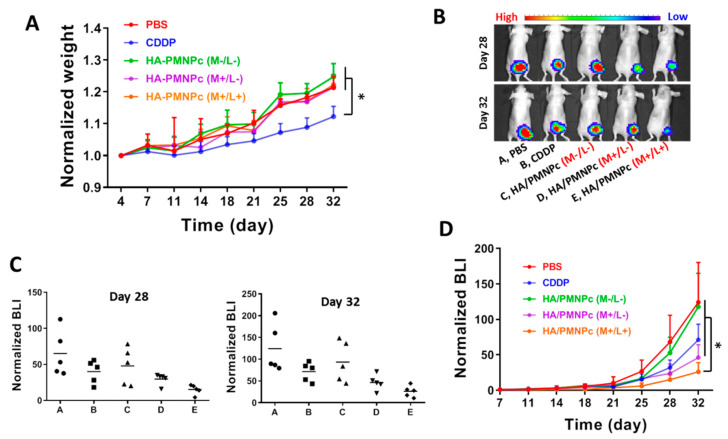
The body weight change during treatment of U87 xenograft tumors in nude mice (**A**). The treatment efficacy was followed from bioluminescence imaging (BLI) by an in vivo imaging system (IVIS) (**B**). The distribution of normalized BLI signal intensity on days 28 and 32 (**C**), and the comparison of normalized BLI values (mean ± SD, n = 5) (**D**). Group: A, PBS; B, CDDP; C, HA/PMNPc (M-/L−); D, HA/PMNPc (M+/L−); E, HA/PMNPc (M/+L+). * *p* < 0.05.

The antitumor efficiency was then performed with U87 tumor-bearing mice. For this purpose, the mice were divided into five groups on day 7 (n = 5 each group), and each mouse was injected with 100 μL solution from the tail vain. The mouse in the control group was injected with PBS; in the CDDP group, injected with a CDDP solution; and in the HA/PMNPc (M−/L−) group, injected with an HA/PMNPc solution (1 mg CDDP/kg body weight dosage). Two additional treatment groups were included at the same CDDP dosage, viz. HA/PMNPc (M+/L−) and HA/PMNPc (M+/L+). The mouse in the HA/PMNPc (M+/L−) group was injected with an HA/PMNPc solution and followed by magnetic guidance with a magnet at the tumor area for 1 h. The mouse in the HA/PMNPc (M+/L+) group was injected with an HA/PMNPc solution, followed by magnetic guidance with a magnet at the tumor area for 1 h and 808 nm laser irradiation for 5 min. As shown in Figure 11A, the weight of nude mice treated with CDDP shows a significant change in the normalized body weight from other groups, possibly from the drug-associated toxicity of free CDDP [77]. The bioluminescence imaging (BLI) with IVIS was used to evaluate the treatment efficacy. As shown from the representative IVIS images on days 28 and 32 in Figure 11B, the BLI signal intensity is the highest in the PBS control group and different treatments result in different changes in the BLI signal intensity. Nonetheless, the HA/PMNPc (M+/L+) group always shows the lowest bioluminescent signal from the U87 tumor cells that can stably express luciferase activity. To quantitatively investigate the antitumor efficacy, all the BLI signal intensity values were standardized to their individual baseline at the time of grouping (day 7), and the normalized BLI is shown for day 28 and day 32 in Figure 11C. The mean value of the normalized BLI intensity is in the order of PBS > HA/PMNPc (M−/L−) > CDDP > HA/PMNPc (M+/L−) > HA/PMNPc (M/+L+). At the end of the observation period (day 32), the value is significantly lower for the HA/PMNPc (M/+L+) group than all other groups (Figure 11D). This indicates the best nanomedicine-based cancer treatment benefit is provided by HA/PMNPc after induction with magnetic guidance and NIR irradiation for dual-targeted chemo-photothermal therapy.

**Figure 12:**
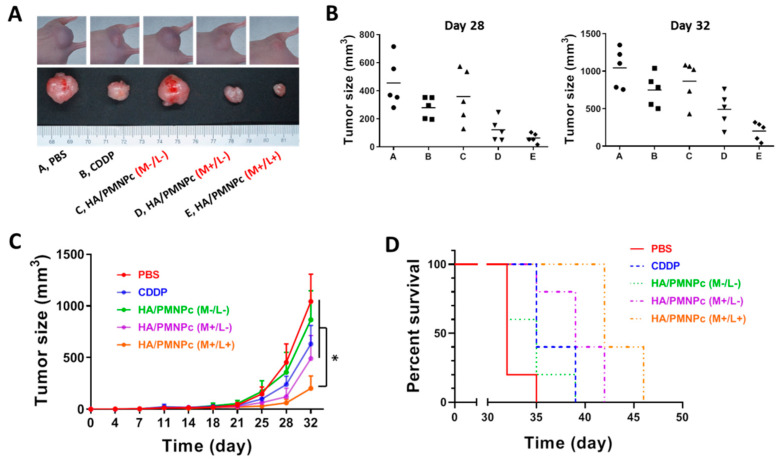
The treatment of U87 xenograft tumors in nude mice was followed by the change in tumor size with the gross view of tumors on day 28 (**A**), the tumor size on day 28 and day 32 (**B**), the change in tumor size (mean ± SD, n = 5) (**C**), and the survival curve (**D**). Group: A, PBS; B, CDDP; C, HA/PMNPc (M−/L−); D, HA/PMNPc (M+/L−); E, HA/PMNPc (M/+L+). * *p* < 0.05.

To confirm the antitumor efficacy from the IVIS as well as to determine the animal survival times, we also use a caliper to measure the tumor dimensions and calculate the tumor volume during the experiment. A general observation of the tumor size on day 28 indicates the CDDP treatment can reduce the tumor size compared with the PBS control, but the HA/PMNPc (M−/L−) group has a larger tumor size than the CDDP (Figure 12A). With magnetic targeting, the HA/PMNPc (M+/L−) treatment can shrink the tumor to a size smaller than the CDDP, which could be further reduced by a combination with NIR laser irradiation in the HA/PMNPc (M+/L+) group. The tumor size from all the animals on days 28 and 32 were included in Figure 12B for comparison. A general trend shown from the change in the mean tumor volume is in line with those observed from the IVIS imaging. Furthermore, the best treatment efficacy provided by the HA/PMNPc (M+/L+) treatment is confirmed with its lowest tumor volume on day 32, which is significantly different from all the other groups (Figure 12C). The animals were observed continuously throughout the experiments and sacrificed when the tumor size reached 1000 mm^3^, from which a survival curve could be constructed (Figure 12D) and the median and average survival times could be calculated (Table 2). It can be seen that the HA/PMNPc (M/+L+) treatment leads to the longest median survival time of the tumor-bearing mice, which also significantly prolongs the average survival time when compared with the other groups. Taken together, the antitumor treatment study with U87 xenografts supports the use of HA/PMNPc (M+/L+) for achieving the best treatment outcomes.

**Figure 13:**
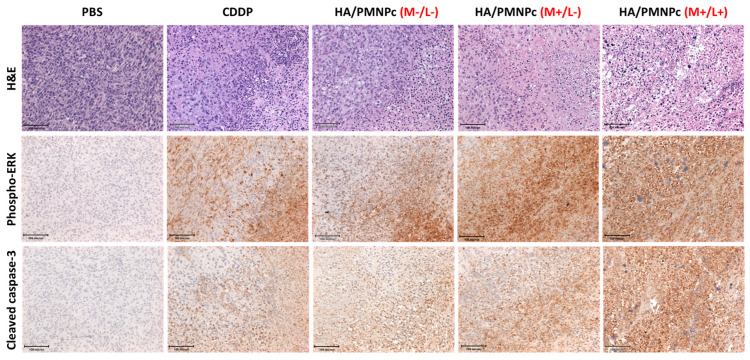
The hematoxylin and eosin (H&E) staining and the immunohistochemical (IHC) staining of phospho-ERK and cleaved caspase-3 in the tumor apoptotic area after different treatments (bar = 100 μm).

Figure 13 is the histological analysis of the tumor sections from the H&E staining and IHC staining. As shown from the H&E staining, uniformly distributed cancer cells without evidence of necrosis are noted in the tumor sample of the PBS (control) group. In the drug-treated groups, the cancer cells show morphological alterations after the apoptosis events, including cell shrinkage, nuclear fragmentation, and the production of apoptotic bodies [78]. The tumor samples taken from the HA/PMNPc (M−/L−) groups demonstrate a much higher cellular intensity than the CDDP group judging from the purple colors from the stained nuclei of the U87 cancer cells. The CDDP group also reveals a higher cell density than both the HA/PMNPc (M+/L−) and HA/PMNPc (M+/L+) groups. Most importantly, the HA/PMNPc (M+/L+) group shows the lowest cell density among all the treatment groups, which is consistent with the results shown from the tumor size and normalized BLI value. From the IHC staining of the hallmark cell apoptosis marker proteins (phospho-ERK and cleaved caspase-3), more apoptotic proteins synthesis was noted from the HA/PMNPc (M/+L+) treatment. Undoubtedly, by showing the most intense brown color, this treatment leads to the highest cell apoptosis rate with the highest level of phospho-ERK and cleaved caspase-3 productions in the cell apoptotic area. The cleaved caspase-3 is an important biomarker to evaluate cancer cell apoptosis, and its expression in the apoptotic tumor area strongly supports that tumor cell death is associated with apoptosis [79]. Similarly, the activation of phospho-ERK in activated stress-related signaling pathways also provides evidence of cell death from apoptosis [80]. The cell apoptosis was found in all the groups except the PBS, where a chemotherapeutic effect is observed from the CDDP or HA/PMNPc treatment. Nonetheless, the HA/PMNPc (M/+L+) treatment leads to the highest cell-killing effect in vivo when guided with a magnetic field and complemented with photothermal therapy. Therefore, this combination photothermal-chemotherapy can limit the growth of a tumor to the highest extent, as shown from the BLI and tumor size measurements (Figure 11 and Figure 12).

## 4. Conclusions

In this study, we coated the CDDP-loaded NIR-responsive PLGA magnetic nanoparticles with HA to prepare the HA/PMNPc, which is successfully used for chemo-photothermal combination cancer therapy both in vitro and in vivo. The OA-modified IOMNP were well encapsulated in the spherical PMNPc nanoparticles, which can be used for magnetic targeted drug delivery. By coating the PMNPc with HA, the HA/PMNPc exhibits a much higher intracellular uptake efficiency than the PMNPc, which allows for the active targeting of U87 cancer cells. In combination with a pH-responsive drug release, the HA/PMNPc can lower the IC50 value when compared with free CDDP, and the cytotoxicity could be further enhanced by irradiation with an NIR laser for chemo-photothermal cancer therapy. Using a xenograft tumor model in nude mice, the mice injected with HA/PMNPc from the tail vein, followed by magnetic guidance and NIR laser irradiation at the tumor site, results in the lowest tumor growth rate and the longest survival time. Overall, the dual-targeting ability as well as the chemo-photothermal therapeutic ability provided by HA/PMNPc endorses its use as an effective treatment modality against tumors from U87 glioblastoma cells.

## Figures and Tables

**Table 1:** The properties of different nanoparticles.

Sample ^1^	Average Diameter (nm) ^2^	PDI ^3^	Zeta Potential (mV)	Crystalline Size (nm) ^4^	Fe_3_O_4_ (%) ^5^	Fe_3_O_4_ (%) ^6^
IOMNP	218.0 ± 10.3	0.13 ± 0.01	22.1 ± 0.2	9.8	100.0 ± 3.1	97.1 ± 1.4
OA-IOMNP	230.7 ± 12.2	0.14 ± 0.02	−18.4 ± 0.5	10.6	90.0 ± 1.3	87.7 ± 2.5
PMNPc	242.2 ± 7.6	0.13 ± 0.02	−25.2 ± 0.3	10.6	11.2 ± 0.1	10.3 ± 0.1
CS/PMNPc	277.2 ± 7.8	0.24 ± 0.01	28.4 ± 0.3	10.3	10.5 ± 0.4	10.1 ± 0.2
HA/PMNPc	337.4 ± 10.8	0.22 ± 0.02	−30.9 ± 1.8	10.5	10.0 ± 0.1	9.9 ± 0.1

^1^ IOMNP: iron oxide magnetic nanoparticles, OA-IOMNP: oleic acid (OA)-coated IOMNP, PMNPc: CDDP-loaded PLGA magnetic nanoparticles, CS/PMNPc: chitosan (CS)-coated PMNPc, HA/PMNPc: HA-coated PMNPc; ^2^ polydispersity index; ^3^ from dynamic light scattering (DLS); ^4^ from X-ray diffraction (XRD); ^5^ from superconducting quantum interference device (SQUID); ^6^ from inductively coupled plasma optical emission spectrometry (ICP-OES).

**Table 2:** The survival times for U87 tumor-bearing mice after different treatments (n = 5).

Group	Median (Days)	Average (Day) ^1^
PBS	32	32.6 ± 1.3
CDDP	35	37.4 ± 2.2
HA/PMNPc (M−/L−)	35	34.6 ± 2.9
HA/PMNPc (M+/L−)	39	39.4 ± 2.9
HA/PMNPc (M+/L+)	42	43.6 ± 2.2 ^α,β,γ,δ^

^1^ Mean ± standard deviation (SD). ^α^ *p* < 0.05 compared with PBS, ^β^ *p* < 0.05 compared with CDDP, ^γ^ *p* < 0.05 compared with HA/PMNPc (M−/L−), ^δ^ *p* < 0.05 compared with HA/PMNPc (M+/L−).

## Data Availability

The data presented in this study are available on request from the corresponding author.

## Supplementary Materials

The following are available online at https://www.mdpi.com/article/10.3390/pharmaceutics15010290/s1, Figure S1: The Fourier transform infrared (FTIR) spectroscopy analysis, Figure S2: The photothermal response of HA/PMNPc, Figure S3: The photothermal conversion efficiency of HA/PMNPc, Figure S4: The photothermal stability of HA/PMNPc.
