# Supplementary material for: Hyaluronic Acid-Modified Cisplatin-Encapsulated Poly(Lactic-co-Glycolic Acid) Magnetic Nanoparticles for Dual-Targeted NIR-Responsive Chemo-Photothermal Combination Cancer Therapy
Source: Pharmaceutics. 2023 Jan 14;15(1):290. doi: 10.3390/pharmaceutics15010290 (PMC9862698; doi:10.3390/pharmaceutics15010290)
Supplement: Supplementary file 1 [file pharmaceutics-15-00290-s001.zip › pharmaceutics-2100035-supplementary.pdf]

Supplementary Materials

Hyaluronic Acid-Modified Cisplatin-Encapsulated Poly(Lactic-co-Glycolic acid) Magnetic Nanoparticles for Dual-Targeted NIR-Responsive Chemo-Photothermal Combina-tion Cancer Therapy

Fourier-Transform Infrared (FTIR) Spectroscopy Analysis

For FTIR analysis of components of HA/PMNPc and all nanoparticles during synthesis of HA/PMNPc, a Bruker Tensor 27 FTIR spectrometer (Billerica, MA, USA) was used. The samples were dried in an oven for 24 h, blended with KBr and ground to fine powder. The transmittance was recorded from 4000 to 400 cm^{-1} at 4 cm^{-1} interval with 16 scans.

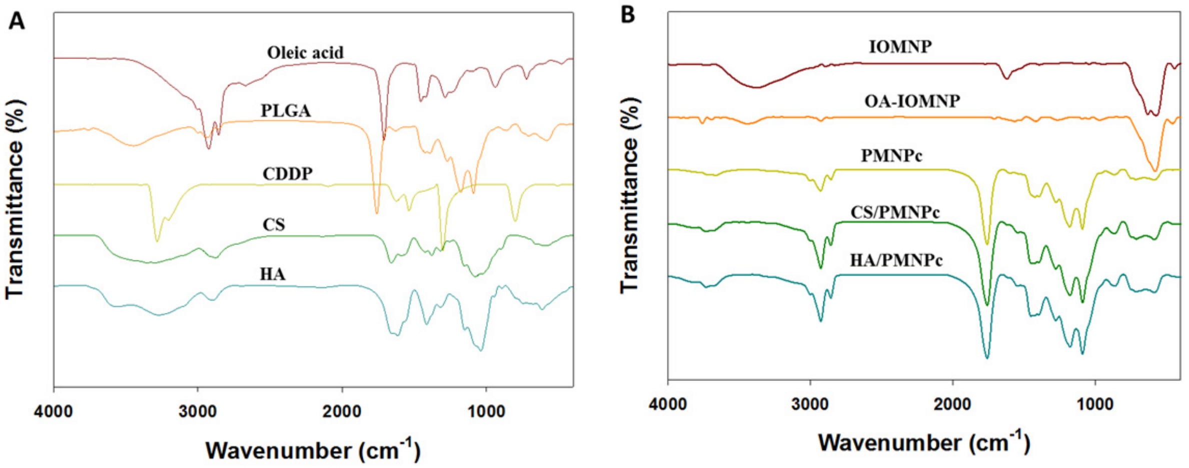

Wavenumber (cm^{-1})	3600-3200	3280	3200	2950	2900	2850	1750-1680	1650	1450-1400	1050	800	640	570	570
Bond	N-H (NH_2) O-H	N-H (NH/NH_3)	N-H (NH/NH_3)	C-H (-CH)	C-H ($-CH_2$)	C-H ($-CH_3$)	C=O (ester/ketone/aldehyde/ carboxylic acid)	N-H (NH_2) C=C (alkene)	O-H (carboxylic acid) C-H ($-CH_2$)	C-O (ester/alcohol)	N-H (NH/NH_3)	Fe-O	Fe-O	Sn-O
Oleic acid	V			V	V	V		V	V					
PLGA	V			V	V	V	V	V	V	V				V
CDDP		V	V								V			
CS	V			V	V			V	V	V				
HA	V	V	V	V		V	V		V	V	V			
IOMNP	V							V				V	V	
OA-IOMNP	V			V	V	V	V	V	V			V	V	
PMNPc	V	V	V	V	V	V	V	V	V	V	V	V	V (550)	
CS/PMNPc	V	V	V	V	V	V	V	V	V	V	V	V	V (550)	
HA/PMNPc	V	V	V	V	V	V	V	V	V	V	V	V	V (550)	

Figure S1. The FTIR analysis of (A) oleic acid, poly(lactic-co-glycolic acid) (PLGA), CDDP, chitosan (CS), hyaluronic acid (HA), and (B) iron oxide magnetic nanoparticles (IOMNP), oleic acid (OA)-coated IOMNP (OA-IOMNP), CDDP-loaded PLGA magnetic nanoparticles (PMNPc), CS-coated PMNPc (CS/PMNPc) and HA-coated PMNPc (HA/PMNPc).

Photothermal Response of HA/PMNPc

The photothermal response of HA/PMNPc was studied by irradiating an aqueous solution of HA/PMNPc (1 mg/mL) in an Eppendorf tube with 808 nm near infrared (NIR) laser at 1.75

W/cm^2. The thermal images were acquired at different time points from tube side using an infrared camera and time-lapsed peak temperature was determined.

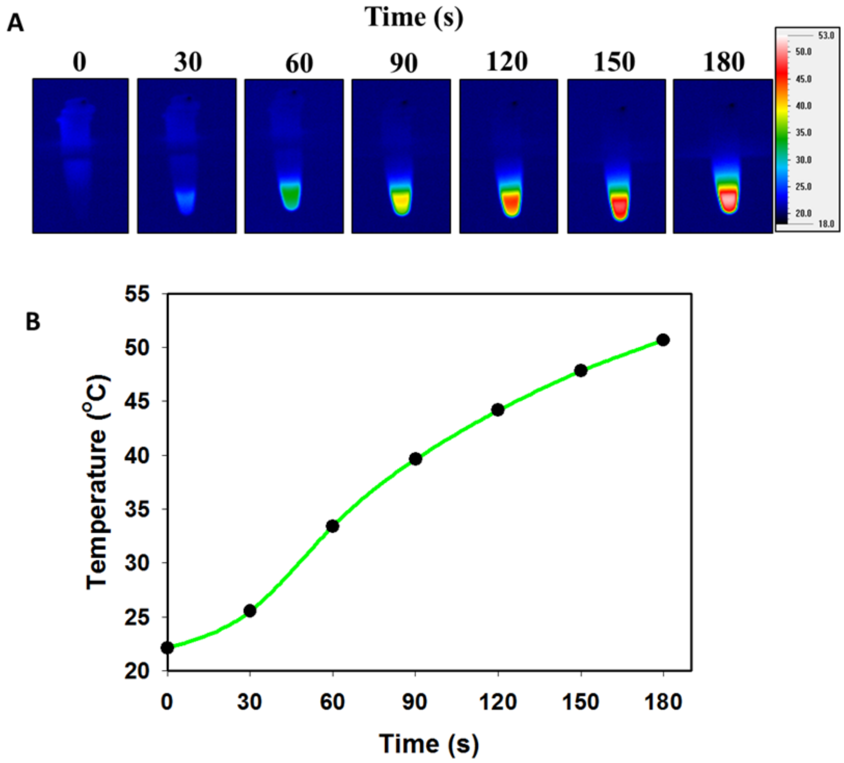

Figure S2. The time-lapsed photothermal images (A) and the peak temperature profiles (B) when an aqueous solution of HA/PMNPc (1 mg/mL) was irradiated by 808 m near infrared (NIR) laser at 1.75 W/cm^2.

Photothermal Conversion Efficiency of HA/PMNPc

An aqueous solution of HA/PMNPc (1 mg/mL) prepared in an Eppendorf tube was irradiated by 808 nm near infrared (NIR) laser at 1.75 W/cm^2. The photothermal conversion efficiency (η) was calculated from equation (1) by monitoring the temperature profile continuously for 360 s with 180 laser on-time and 180 s laser off-time.

$$\eta = \frac{hS\,(T_{max} - T_{sur}) - Q_0}{I\,(1 - 10^{-A_{808}})} \qquad (1)$$

The h is the heat transfer coefficient and S is the tube surface area. T_{max} and T_{sur} represent the maximum and surrounding temperatures, respectively. Q_o represents the heat absorbed by the tube (assumed to be 0), I represents the power of the laser, and A_{808} represents the solution absorbance at 808 nm wavelength. The dimensionless parameter θ is defined by equation (2).

$$\theta = \frac{T_{sur} - T}{T_{sur} - T_{max}} \qquad (2)$$

The time constant (τ_s) from the cooling curve was calculated from equations (3) and (4).

$$-\ln(\theta) = \frac{t}{\tau_s} \qquad (3)$$

$$hS = \frac{mC}{\tau_s} \qquad (4)$$

Where m is water mass and C is water heat capacity.

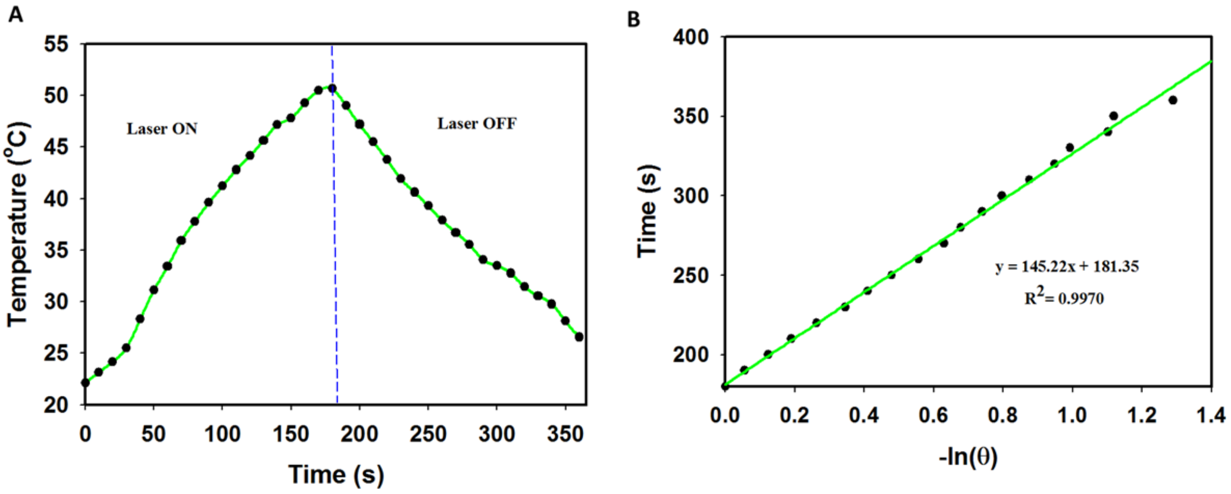

T_{max}	T_{sur}	τ_s	M (g)	C (J/g)	hS (mW/℃)	A_{808}	I (mW)	η (%)
50.66	22.13	145.22	0.28	4.2	8.09	0.316	1750	25.53

Figure S3. (A) The heating and cooling curves after irradiation an HA/PMNPc aqueous solution (1 mg/mL) with 808 m near infrared (NIR) laser at 1.75 W/cm for 3 min. (B) The time constants (τ_s) was determined from the slope of the cooling curve, from which the photothermal conversion efficiency η could be calculated

Photothermal Stability of HA/PMNPc

To determine photothermal stability, an aqueous solution of HA/PMNPc (1 mg/mL) was irradiation with 808 nm NIR laser (1.75 W/cm^2) for 360 s with 180 laser on-time and 180 s laser off-time. The process was repeated for 5 cycles using the same solution. The thermal images were acquired with an infrared camera at different time points and the time-lapsed peak temperature profile was determined during the five cycles.

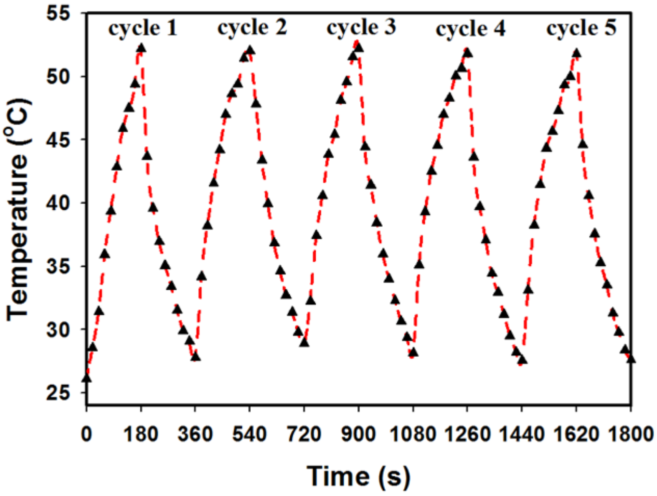

Figure S4. The peak temperature profiles after repeated irradiation of an aqueous solution of HA/PMNPc (1 mg/mL) with 808 m near infrared (NIR) laser at 1.75 W/cm for 5 cycles with 180 laser on-time and 180 s laser off-time in each cycle.
